# Notes and News

**Published:** 1887-03-05

**Authors:** 


### NOTES AND NEWS.

COLLECTED AND COMMUNICATED.

LORD BRABAZON has consented to become a trustee of the Morley House Convalescent Home, St. Margaret's, Dover.

THE Arbroath Infirmary has surmounted its financial difficulties, and has nearly £200 in hand. A legacy of £100 has been bequeathed to this useful hospital by the late Misses Marnie.

THE late severe weather has exerted its baneful influence in the matter of chest disease. During the past three months the whole of the 321 beds comprised in the two buildings of the Brompton Hospital have been fully occupied, while about 290 applicants are now anxiously awaiting their turn for admission.

THE annual festival dinner in aid of the Dental Hospital of London, Leicester Square, has been fixed to take place at the Hotel Métropole, on Thursday, 10th inst. Sir James Paget, F.R.S., will preside. The annual festival dinner in aid of the Royal National Hospital for Consumption, Ventnor, will be held at the same place on April 19th. The Duke of Cambridge will occupy the chair.

MADAME MARIE ROZE, who has repeatedly shown her warm-hearted sympathy with the sick poor, accepted a few days since an invitation from the Governors to visit the new Jaffray Hospital at Birmingham, a most useful institution, which acts as a kind of suburban adjunct to the General Hospital. Madame Roze visited nearly all the wards and spent a considerable time in conversation with the inmates. Before leaving, Madame Roze sang several songs to those of the patients who were well enough to assemble and hear her.

AN entertainment of a highly successful character was recently given at Lowther Lodge, Kensington Gore, in aid of the East London Nursing Society, and Mrs. Walsham How's Industrial Home. The Princess Christian of Schleswig-Holstein honoured the performance with her presence. The artistes included Signor Romili, Mr. and Mrs. Henschel, Mrs. Stirling, Mrs. Kendal, Signor Piatti, and Mr. Eric Lewis. Mr. A. W. Lacey, 49, Philpot Street, Commercial Road, E., is the secretary of the East London Nursing Society, and Mr. T. E. T. Ripley, 37, Stanhope Gardens. S.W., of Mrs. How's Home.

THE oldest dispensary in London is the Royal General in St. Bartholomew Close. It was founded in Shaftesbury House, Aldersgate Street, just ten years after George III became king, so that it is now 117 years old. Last year, no fewer than 11,050 cases were dealt with by the medical officers, and 2,178 visits were paid by the doctors to 546 patients at their own homes. The Governors are anxious, during this Jubilee year, to clear off the debt of £5,000 which at present exists on account of the loan which they were compelled to raise, to acquire the freehold of the dispensing building.

ON Thursday evening, the 17th ult., a drawing-room entertainment was given at the Cancer Hospital, Brompton, for the amusement of the in-patients. Dr. Marsden and several of the staff were present. Mr. H. G. Clarence, a most clever exponent of the *art magique*, opened the entertainment with a number of surprising sleight-of-hand tricks; these were followed by his "living marionettes"; and then came some marvellous examples of thought-reading. The entertainment concluded with the sensational illusion of the "Vanishing Lady," Mrs. Clarence, the "baseless fabric of a vision," reappearing, to the astonishment of the patients and their friends, at the further end of the room with a dove on her finger. Dr. Marsden, in thanking Mr. Clarence for his excellent performance, said that he had never spent a more charming and delightful evening in that room.

THERE is, perhaps, no hospital in the world so widely favoured by the crowned heads of Europe as the Seamen's Hospital at Greenwich. For the lengthened period of half a century our own Queen has subscribed 100 guineas a year, while the Emperors of Russia, Germany, and Austria, and the Kings of Italy, Holland, and Portugal, and the Governments of Russia, Norway, Finland, Sweden, Spain, Holland, and China, all subscribe. Sir Andrew Clark, Bart., M.D., recently paid a visit of inspection to the hospital, and at the annual court of the Society last week, he expressed, in very eloquent terms, his sense of admiration of the splendid efficiency to which this institution has now attained. The multiplicity of appliances and conveniences for the relief of the sick specially attracted Sir Andrew's attention. The hospital, which last year treated no less than 2,095 in-patients and 5,359 out-patients, was established in 1821, on a very small scale, on board the *Grampus;* thence it was transferred to the *Dreadnought*—a name by which it is still familiarly known—and in 1870 it was removed on shore, to the present extensive premises, formerly the Infirmary of Greenwich Hospital. A few years ago a dispensary in Well Street, London Docks, and more recently one at Gravesend, have been attached to it.

WHAT is the least number of persons that can be said to constitute "a meeting"? We have probably most of us heard of the Irishman who held a meeting in support of Home Rule, "all by himself," and passed two "wholly unanimous resolutions." At a recent annual meeting of the subscribers of a hospital in the North of England, owing probably to the inclement weather, only three persons, the secretary included, put in an appearance. "A" took the chair, "B," the secretary, read the annual report, and "C," the "meeting," listened to it. "A" moved its adoption, while "C" seconded; then "A" moved a vote of thanks to the absent medical officers, and "B" seconded. But then "the meeting" could not break up without a vote of thanks to the "chair," and so there was nothing to do but for "C" to propose and "B" to second this *pro formâ* bit of business. The thought crosses our mind—what would have happened if "C" had failed to attend!

---

THE following vacancies are announced this week, the amount of the salary being placed in each case after the name of the institution:—

**Honorary Medical Officers.**

Infirmary for Consumption, Margaret Street, W. Physician-in-Ordinary, three Visiting Physicians, and one Surgeon.
National Hospital for the Paralysed. Laryngologist.
North-West London Hospital. Assistant Physician.

**Resident Medical Officers.**

Ashton-under-Lyne Infirmary. House Surgeon. Doubly qualified.—£80.
Birmingham Eye Hospital. House Surgeon. Qualified.—£100.
Boscombe Infirmary. One. Qualified.—£60.
Chelsea Hospital for Women. One. Registered.—£60.
Nottingham Hospital. Junior. Doubly qualified.—£100.
Paddington Workhouse Infirmary. Assistant Superintendent and Dispenser. Qualified.—£100.

**Non-resident Medical Officers.**

City of London Hospital for Diseases of the Chest. Pathologist. Registered.
Chelsea Hospital for Women. Dispenser. Qualified.—£70.
Owens College, Manchester. Senior Demonstrator in Physiology.—£150.

**Matron.**

St. John's Hospital, Northampton. Matron.—£40.

**Trained Nurses.**

Great Yarmouth Temporary Hospital. One.
Hertford Infirmary. One Assistant.—£16 to £20.
Westhulme Fever Hospital, Oldham. One.—£25.
Western Dispensary, Marylebone. One.—£20.

**Probationers.**

Hospital for Women, Lupus Street. One.—Fee, £1 1*s.* a week.
Kent Nursing Institution, West Malling. Three.
Westhulme Hospital, Oldham. One.—£16.

---

IN 1885, for the first time for ten years, the receipts of the Stroud General Hospital fell short of the expenditure, and the unfavourable balance was not wiped off in 1886, so that the institution starts the present year over £40 in debt. The Hospital Sunday Collection exhibited an increase of £48 5*s.* 0*d.*, but this was solely due to one generous contributor who placed £50 in the hospital-box on that day. The total income was £24 less than in 1885, but considerable economy in expenditure has been effected, and there is a total diminution under this head of £148 15*s.* 8*d.* Fourteen subscribers have discontinued their support, but as seventeen new ones have been received, there is a slight gain from this important source of income. The workpeople's contributions show a falling off of £36 9*s.* 1*d.* as compared with 1885, and of £105 6*s.* 1*d.* as compared with 1884. Much regret is expressed at the retirement from active duty of Dr. Paine, after thirty-five years of regular attendance at the Hospital.

THE DUKE OF CAMBRIDGE has kindly promised to take the chair at a festival which is to be held at the Hotel Métropole on Friday, 6th May, in commemoration of the 20th anniversary of the Victoria Hospital for Children, Chelsea. This admirable institution occupies the site—and indeed the house—memorable as the residence of the Goughs. In 1866, Gough House, as it was called, was converted into the Victoria Hospital for Sick Children, being one of the very first institutions of the kind devoted entirely to the diseases and accidents of childhood. In 1876 a Convalescent Home in connection with the hospital was opened at Churchfields, Margate.

---

THE Committee of the Eastern Counties Children's Convalescent Home have been enabled, through the generosity of some kind friends, to purchase the block of buildings where the Home is now housed, together with the adjacent residence. This addition will much enlarge the usefulness of this admirable institution, which is not only a curative, but also a preventive agency. The Mayoress of Yarmouth takes a deep interest in the welfare of the Home.

---

THE British Hospital at Port Said, to which we recently drew attention, is now in course of erection. Over £2,000 has already been collected, but the sum required for the building of the hospital and its complete equipment is estimated at £4,000. The site of the hospital has been given by the Egyptian Government, and is that on which the National Memorial to the late General Gordon was to have been reared. The hospital, though it will afford relief to sick mariners of all nations, will be entirely under British administration, and it will be nursed by English nurses. With our rapidly increasing traffic through the Suez Canal, bound for India, China, the Australian Colonies, etc., the need of such a refuge for sick seamen is more and more felt, and it is earnestly to be hoped that its promoters will be able to open it free from the incubus of debt. Sir J. Stokes, K.C.B., of Good Rest, Hayward's Heath, Sussex, will gladly supply information about this hospital.

---

WE recently drew the attention of our readers to a proposed hospital for the poor mine women of Cornwall. At the present time there are 874 females working in the mines, and 433 in the tin streams, at wages averaging from 25*s.* to 35*s.* a month. At an influential meeting recently held at Redruth, a committee was appointed to select a site for the erection of a suitable hospital for the women working in the mines and tin streams. It is hoped to work the institution on lines similar to those of the Miners' Hospital. There is a strong feeling in Cornwall in favour of opening the hospital for poor women generally, but at present it is not considered advisable to go beyond the class originally intended. Mr. G. L. Basset, J.P., has promised to pay one half of the initial cost of the building, and to give £100 a year, and Mr. J. C. Williams has volunteered a similar annual contribution.

THE following sums have been recently received by the various Medical Charities, the name of the donor or the source from which they were obtained being given in each instance after the name of the Institution :—

**Donations.**

Chelsea Hospital for Women, Fulham Road. L. S., £20. Mr. J. Vernon Watney, £10 10*s*. The Company of Leathersellers, £10 10*s*.
Charing Cross Hospital. Dr. Montagu Lubbock, £21. Misses Noble, £10.
North London Hospital. Mrs. Elizabeth R. Müller, £31 10*s*. Rev. A. J. Harvey, £50.
Hospital for Diseases of the Skin, Golden Square. Messrs. Baring Brothers, £10 10*s*.
Surgical Aid Society. The Company of Leathersellers, £50. The Company of Grocers, £21.
National Hospital for Consumption, Ventnor. The Company of Fishmongers, £52 10*s*.
North-Eastern Hospital for Children. The Company of Leathersellers, £10 10*s*.
Hospital for Women, Soho Square. The Company of Leathersellers, £10 10*s*.
Dental Hospital of London. Mr. T. Hayter Lewis, F.S.A., £10 10*s*.
West London Hospital. J. B., £100 (Jubilee Offering). The Company of Leathersellers, £21. Mr. Watney, £10. The Hammersmith Vestry, £21.
Morley House (Hospital Saturday) Convalescent Home, St. Margaret's, Dover. The Mercers' Company, £31 10*s*.
North London Hospital. Mrs. A. Spragoe, £10 10*s*. People's Fund in aid of the Hospital, £85,
Surgical Aid Society. Company of Goldsmiths, £50.
Royal Hospital for Incurables, Putney. Miss Johnstone, £200 (Jubilee Offering).

**Collections.**

West London Hospital. Kensington Parish Church, £68 5*s*. 6*d*.; and for Chaplain's fund, £20.

**Legacies.**

Metropolitan Convalescent Institution, Walton-on-Thames. Mrs. A. M. Benthall, £100 Trustees of Prison Charities, £105. Mr. John McKinnell, £45.
Hospital for Consumption, Brompton. Miss Lloyd, £20. Mrs. Mills, £350. Captain W. B. Phillimore, £300. Mrs. Sinclair, £200.
Morley House (Hospital Saturday) Convalescent Home, St. Margaret's, Dover. Mr. Samuel Morley, £100.

---

WE have frequently of late had to comment with regret upon the inability of many of the charitable institutions in various parts of the country to cope not only with their increasing but even with their normal pecuniary wants. A fresh instance of this lamentable falling off in income is furnished by the Macclesfield Infirmary, the fourteenth annual report of which has recently been sent us by the Secretary, Mr. G. H. Corbishley. The expenditure for 1886 amounted to nearly £3,082, whilst the receipts were under £2,956, thus leaving a deficit of £126 chargeable to the current account. The Hospital Sunday collection was the smallest on record, and the Governors "deplore so serious and so needless a loss to an important charitable institution," a loss which they attribute to a want of unanimity on the part of the clergymen and ministers in the selection of a day for the collection. Last year the principal chapels of the district, from which the largest sums are usually obtained, omitted to have collections on Hospital Sunday, and though these were subsequently held, there can be little doubt that a far better result might have been obtained if the wiser course had been closely adhered to. The annual increase in the number of patients at the Infirmary was maintained in 1886, there having been 112 in- and 514 out-patients more than in the preceding year, whilst 443 casualty cases were treated, as against 369 in 1885. A notable item under the head of "disbursements" is the sum of £70 16*s*. 6*d*. for stimulants, being £25 16*s*. 0*d*. more than in the previous twelve months. This increase is explained as the result of demands for grave cases of illness made by the medical officers, and is not to be attributed to extravagance on the part of the management. The Thornycroft Convalescent Fund has provided for 90 patients, who have been received for varying periods at convalescent homes in Southport, Buxton, and Cheadle. £140 is required to make up the endowment of this fund to £1,000, an object of considerable importance, as the interest arising from the portion now invested cannot be made available until the stipulated total is complete, and even then annual subscriptions will be needed to satisfactorily carry on this important branch of the infirmary work.

---

IT would be well if the speech of the Lord Provost at the annual meeting of the Glasgow Royal Infirmary could be pressed upon the earnest attention of every resident of the town. One thing is evident, and that is, that, much as the people of Glasgow do, they do not perform their whole duty towards the oldest hospital in the city. Last year, while the expenditure amounted to £24,637, the ordinary revenue was only £18,183, leaving a deficit of £5,854. An institution which has carried on its good work for upwards of three-quarters of a century, and is so largely sought after by the poor of the town, is surely worthy of adequate support. Wealthy men ease the burden of their conscience by "an annual dole of the stereotyped guinea," as the Lord Provost aptly termed it. In this great and prosperous city the annual subscribers of five guineas and upwards number barely one hundred. If it had not been for the extraordinary revenue of the Infirmary—the legacies of the year amounting to £6,145—this grand Scottish charity would have been in a sad predicament. But legacies are a very precarious source of income, and one to whose tender mercies an institution which has deserved so well of the people as the Glasgow Royal Infirmary, ought assuredly not to be left.

---

THE matron of a provincial cottage hospital recently addressed a letter to the Editor of THE HOSPITAL, asking for his opinion with regard to the expenditure at her institution, of which details were enclosed. Her committee were disposed to think that reduction in various items could be made, whereas our correspondent could not see how, if the standard of efficiency was to be maintained, any further economy could be practised. The accounts in question were forthwith compared with those of another cottage hospital, which we knew to be very inexpensively managed, and it was found that the disputed items were extremely moderate. Indeed, the sum of £5 10*s*. for "Surgery and Drugs," for the use of nearly ninety patients, and extending over a period of 3,450 days, seemed incredibly small, as did also the amounts paid as wages to the under servants. These views were duly communicated to our correspondent, and we shall be glad to hear whether the production of her accounts has proved as satisfactory to the committee of the institution in question as it has been to the management of this paper.

